# A quantitative real-time approach for discriminating apoptosis and necrosis

**DOI:** 10.1038/cddiscovery.2016.101

**Published:** 2017-01-23

**Authors:** Asha Lekshmi, Shankara Narayanan Varadarajan, Santhik Subhasingh Lupitha, Deepa Indira, Krupa Ann Mathew, Aneesh Chandrasekharan Nair, Mydhily Nair, Tilak Prasad, Hari Sekar, Anurup Kochucherukkan Gopalakrishnan, Abitha Murali, Thankayyan Retnabai Santhoshkumar

**Affiliations:** 1 Cancer Research Program-1, Rajiv Gandhi Centre for Biotechnology, Thiruvananthapuram, Kerala, India

## Abstract

Apoptosis and necrosis are the two major forms of cell death mechanisms. Both forms of cell death are involved in several physiological and pathological conditions and also in the elimination of cancer cells following successful chemotherapy. Large number of cellular and biochemical assays have evolved to determine apoptosis or necrosis for qualitative and quantitative purposes. A closer analysis of the assays and their performance reveal the difficulty in using any of these methods as a confirmatory approach, owing to the secondary induction of necrosis in apoptotic cells. This highlights the essential requirement of an approach with a real-time analysis capability for discriminating the two forms of cell death. This paper describes a sensitive live cell-based method for distinguishing apoptosis and necrosis at single-cell level. The method uses cancer cells stably expressing genetically encoded FRET-based active caspase detection probe and DsRed fluorescent protein targeted to mitochondria. Caspase activation is visualized by loss of FRET upon cleavage of the FRET probe, while retention of mitochondrial fluorescence and loss of FRET probe before its cleavage confirms necrosis. The absence of cleavage as well as the retention of mitochondrial fluorescence indicates live cells. The method described here forms an extremely sensitive tool to visualize and quantify apoptosis and necrosis, which is adaptable for diverse microscopic, flow cytometric techniques and high-throughput imaging platforms with potential application in diverse areas of cell biology and oncology drug screening.

## Introduction

Apoptosis or programmed cell death is a highly conserved cell death mechanism and has a significant role in embryonic development as well as in the initiation and progression of several diseases. Increased apoptosis is observed in neurodegenerative, autoimmune, inflammatory and cardiovascular diseases.^1,2^ Similarly, loss of apoptosis is a common phenomenon in most of the chemotherapy-resistant cancers.^3–5^ Therefore, the induction of apoptosis in the target cancer cells is the therapeutic goal for any cancer therapy. Consequently, anticancer drug discovery process begins with assays meant for identifying apoptosis-inducing agents. Another form of cell death called necrosis is not preferred as an anticancer strategy as it is nonspecific in nature and manifests with deleterious release of toxic components into the extracellular milieu triggering an inflammatory response. It has been continuously emphasized that drugs that eliminate cancer cells primarily through apoptosis, without the involvement of necrosis, are good cancer drugs, due to the lack of inflammatory responses. Further, necrotic cells are not efficiently cleared by macrophages.^6^ These facts ascribe to the significance of better conclusive methods to distinguish apoptosis and necrosis for cell biology studies and cancer drug screening application.

Most of the signaling involved in apoptosis is well characterized and diverse methods have evolved for determining programmed cell death. The widely used method for apoptosis detection involves analysis of Annexin-V binding on cell membrane, DNA fragmentation, mitochondrial membrane potential loss, mitochondrial permeabilization, Bax activation and caspase activation analysis using fluorogenic substrates.^7,8^ Most of these assays are highly powerful techniques for discriminating apoptotic cells from live cells. However, these assays as such are not useful for discriminating necrotic cell death from apoptosis because of multiple reasons. The popular dye-based approach used for discriminating apoptotic and necrotic cell is based on Annexin-PI staining. Annexin-V exposure, even though considered as an early event of apoptosis, had been initially characterized in necrotic cells.^9^ Likewise, it is well established that most apoptotic cells later shift to necrotic status owing to membrane permeability.^10,11^ This makes it very difficult to ascertain the nature of cell death signaling from snapshot events of analysis using this assay. Another major drawback of these assays is the inability of the late apoptotic cells to bind to Annexin-V if the membrane structures are damaged.

A prominent feature of apoptosis is mitochondrial permeabilization and subsequent caspase activation.^12^ Similarly, a major event that marks the necrotic cell is lack of caspase activation and an increase in membrane permeability.^13^ While imaging caspase activation using FRET-based method to detect apoptosis,^14–16^ we observed that a few cells release the soluble cytosolic caspase sensor probe without any ratio change that is readily detected with diverse imaging platforms. Based on this, we describe a sensitive and confirmatory real-time assay for discriminating apoptosis and necrosis.

## Results

### Loss of FRET probe readily identifies necrotic cells

**Figure 1:**
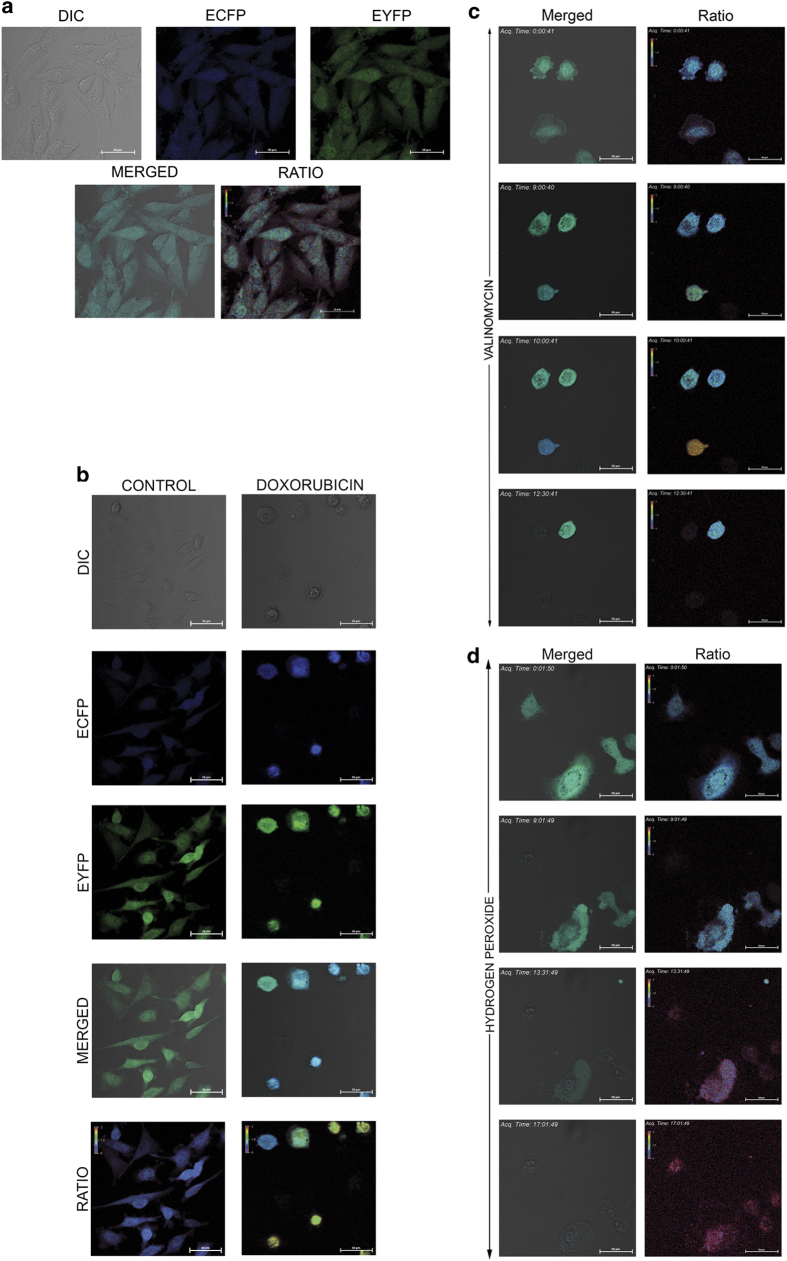
(**a**) U251 cells stably expressing the ECFP-DEVD–EYFP FRET probe (U251 caspase sensor) were developed as described. The microscopic images in brightfield and fluorescence channels are shown. (**b**) U251 caspase sensor cells were treated with doxorubicin (200 ng/ml) for 24 h. The ECFP–EYFP FRET channels and merged image of ECFP/EYFP channels are shown along with the DIC image. The respective image for the untreated control is also shown. (**c**) U251 caspase sensor cell line grown in 96-well glass-bottom plates were exposed to valinomycin (5 *μ*M). The real-time imaging for caspase activation was evaluated using microscope as described in the 'Materials and Methods'. The merged images of DIC/ECFP/EYFP and ratio are shown from indicated time points. (**d**) U251 caspase sensor cell line grown in 96-well glass-bottom plates were exposed to H_2_O_2_ (2.5 mM). The real-time imaging for caspase activation was evaluated using microscopy as described in the 'Materials and Methods'. The merged images of DIC/ECFP/EYFP and ratio are shown from indicated time points.

We have previously described an image-based high-throughput method for detecting caspase activation and for anticancer drug screening using stable cells expressing FRET-based genetically encoded sensor.^15^ This probe consists of donor fluorophore ECFP and acceptor fluorophore EYFP joined with an activated caspase-specific amino acid linker ‘DEVD’. In the present work, we developed neuroblastoma cell, U251 stably expressing caspase sensor as described previously. For easy and uniform imaging purposes, single-cell colonies with homogenous expression of the probe were expanded and used for the current study (Figure 1a). For validation of the cell line for caspase activation detection, wide-field microscopy with single excitation for ECFP and dual emission of ECFP and EYFP was collected in ratio mode using an automated fluorescence microscope. A representative image of donor and acceptor fluorescence of control cells and cells treated with doxorubicin is shown in Figure 1b. As shown in the figure, cells with activated caspases were easily distinguished with an increase in donor fluorescence and decrease in acceptor florescence (measured with a change in ECFP/EYFP ratio). We also noticed 10–15% of cells treated with doxorubicin had lost fluorescence and these cells depicted morphologic features indicative of necrosis such as vacuolization, enlarged cell size, membrane alterations and so on. This suggests that these cells lost the soluble genetically encoded FRET probe probably due to cell membrane permeability changes associated with necrosis. This could either be due to primary necrosis or the secondary induction of necrosis after caspase activation. To distinguish these two possibilities, we exposed the cells with valinomycin followed by time lapse imaging, so that the fate of each cell could be temporally analyzed to distinguish apoptotic cells from cells acquiring necrotic phenotypes after caspase activation. Representative snap shots from the real-time imaging is shown in Figure 1c. (The full sequence is given as Supplementary Video 1). As seen from the video and image, caspase activation is readily visible with increase in blue fluorescence (donor) and subsequent increase in ratio. Also, some of the cells showed necrotic morphology without ratio change (primary necrosis) and a few cells showed sudden release of the transgene after its cleavage (secondary necrosis).

### Caspase sensor cells expressing Mito-DsRed distinguishes apoptosis and necrosis

**Figure 2:**
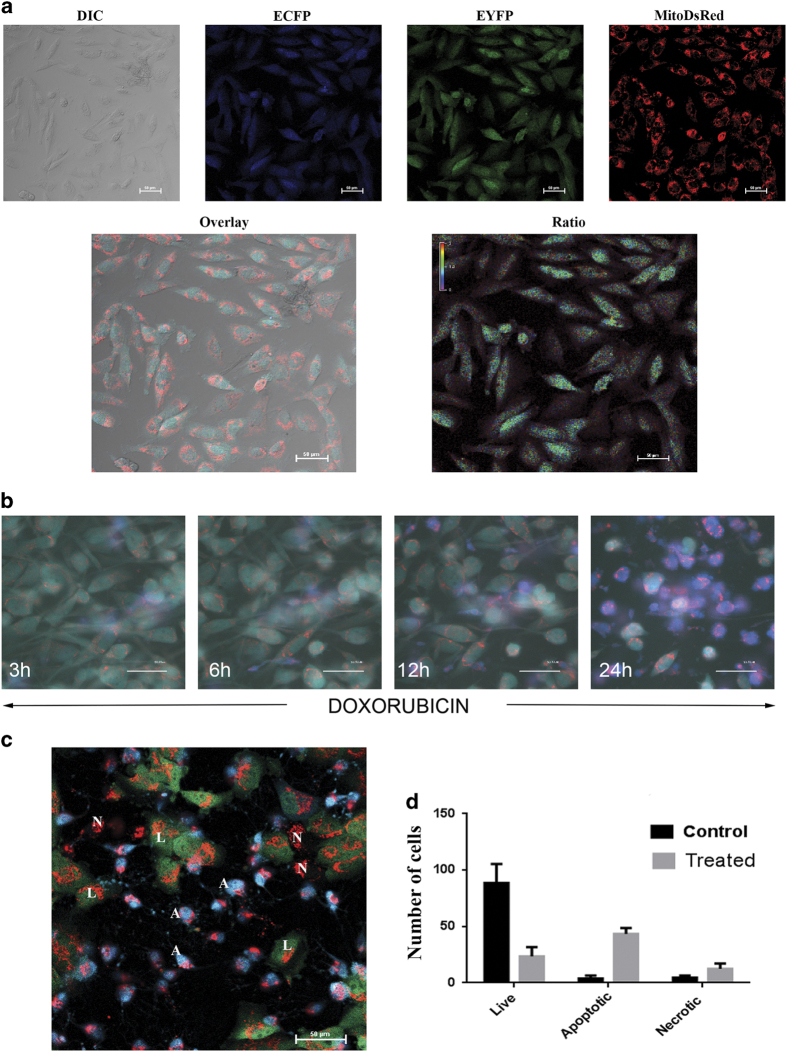
(**a**) U251 caspase sensor cell line was transfected with DsRed targeted at mitochondria to generate stable cells expressing both the probe, U251 DEVD Mito Ds cells. Representative images in brightfield, ECFP, EYFP, DsRed channels and ratio mode of the stable cells are shown. (**b**) U251 DEVD Mito Ds cells were treated with caspase activating compound doxorubicin (200 ng/ml). The real-time imaging for caspase activation and Mito-DsRed fluorescence was carried out using fluorescence microscope at an interval of 10 min as described in the 'Materials and Methods'. The snapshot of merged channels of ECFP/EYFP/DsRed at 3 h, 6 h, 12 h and 24 h of treatment is shown. The caspase activation is reflected from the FRET loss and subsequent increase in blue channel fluorescence. (**c**) Images representing three distinct cell populations identified after doxorubicin (200 ng/ml) treatment of U251 DEVD Mito-DsRed cell: apoptotic cells with ECFP/EYFP ratio change and retaining red fluorescence, necrotic cells without any ECFP/EYFP fluorescence and retaining red fluorescence and live cells with intact ECFP/EYFP probe without ratio change and retaining red fluorescence. In the image, 'A' represents apoptotic cells, 'N' represents necrotic cells and 'L' represents the live intact cells. (**d**) U251 DEVD Mito Ds cells were either untreated or treated with caspase activating compound for 24 h. The cells were imaged with fluorescence microscope for ratio imaging and DsRed channel. The cells with caspase activation and necrosis were identified on the basis of ratio change and DsRed fluorescence as described. The quantitative necrosis and apoptosis from three different independent experiments are shown in the graph. The results represented are mean±S.D. (*n*=3).

The above-described results substantiate that necrotic cells lose the soluble FRET probe because of membrane permeabilization. Therefore, we hypothesized that if a stable non-soluble fluorescent probe is simultaneously transfected into cells along with the FRET probe, necrosis and apoptosis can be distinguished easily by using fluorescence live cell imaging. A better method of expressing non-soluble fluorescent tags is to target the fluorophore to membranous organelles. Based on this, we stably introduced DsRed targeted at the mitochondria that can be easily imaged simultaneous with ECFP–EYFP. Single-cell clones expressing homogenous levels of both probes were developed for imaging and quantification purposes (Figure 2a). The cells expressing this probe were subsequently exposed to apoptosis-inducing drug doxorubicin followed by real-time imaging for 24 h. A representative merged channels of ECFP/EYFP FRET and DsRed at 3 h, 6 h, 12 h and 24 h is shown in Figure 2b. The caspase activation is well reflected from the increase in donor fluorescence with no change in Mito-DsRed. None of the cells turned to necrosis even after 8–10 h of caspase activation in doxorubicin-treated cells (full sequences of images are given as Supplementary Video 2). The Figure 2c shows that three distinct cell populations can be easily identified using this tool; apoptotic cells with ECFP/EYFP ratio change and retaining mitochondrial red fluorescence, necrotic cells without any ECFP/EYFP fluorescence and retaining red fluorescence and live cells with intact ECFP/EYFP probe without ratio change and retaining red fluorescence. Different forms of cell death in the treated samples were quantified and represented in Figure 2d.

### Identification of apoptosis/necrosis by confocal and HTS imagers

**Figure 3:**
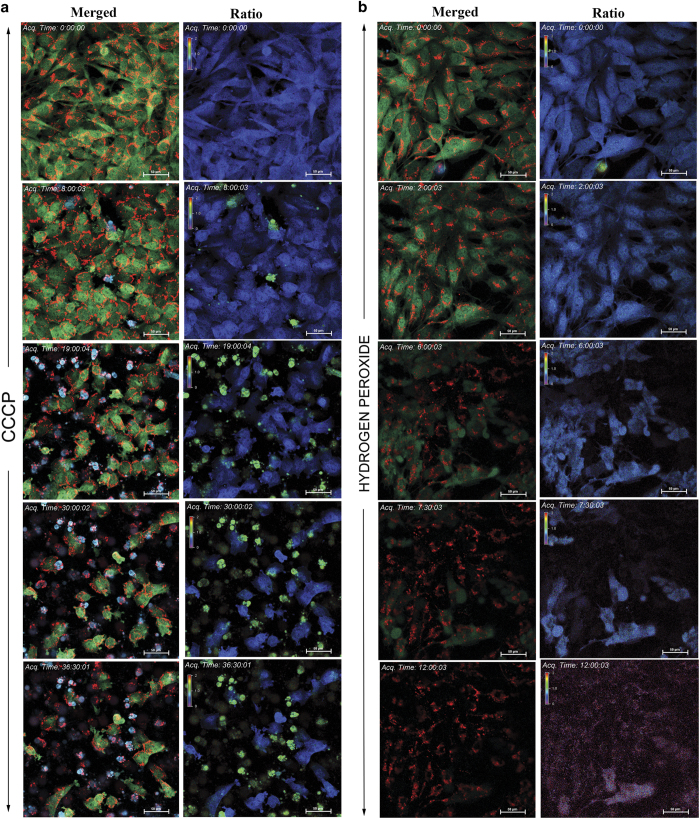
(**a**) U251 DEVD Mito Ds cells were treated with CCCP (5 *μ*M). The real-time imaging for caspase activation and Mito-DsRed fluorescence was carried out using confocal microscopy at an interval of 15 min as described in 'Materials and Methods'. The representative ECFP/EYFP ratio images and merged channels of ECFP/EYFP/DsRed are shown from indicated time points. (**b**) U251 DEVD Mito Ds cells were treated with necrosis-inducing agent H_2_O_2_ (2.5 mM). The real-time imaging for caspase activation and Mito-DsRed fluorescence was carried out using confocal microscopy at an interval of 15 min as described in 'Materials and Methods'. The representative ECFP/EYFP ratio images and merged channels of ECFP/EYFP/DsRed are shown from indicated time points.

We have exposed the cells to H_2_O_2_ and CCCP as described above followed by real-time imaging of ratio change and DsRed at an interval of 15 min using confocal microscope. A representative time lapse image of selected time points from CCCP-treated sample is shown in Figure 3a. The full sequence from the time lapse imaging is shown in Supplementary Video 3A and 3B. CCCP induced FRET loss starting from 8 h of treatment and the time lapse imaging readily identified the cells that showed indication of necrosis after caspase activation. Consistent with the previous results, some of the cells showed primary necrosis. However, as shown in the Figure 3b, subsequent to H_2_O_2_ treatment, cells started to lose the FRET probe from 4 h of treatment without ECFP/EYFP ratio change (i.e., no caspase activation) but all these cells retained the red mito-fluorescence for a prolonged time indicating necrosis. As expected, none of the cells showed indication of apoptotic cell death. The full sequence is given in Supplementary Video 4A and B. Overall, the cell line proved as an extremely sensitive tool for distinguishing apoptosis and necrosis in real time at single-cell resolution.

Additional representative figures for selected drugs and video are shown in Supplementary Figure 1 and Video 5. Consistent with the wide-field microcopy, confocal imaging also proved as a valuable approach for distinguishing necrosis from apoptosis using the developed imaging tool. Several common antitumor agents were used for understanding the nature and dynamics of cell death. In the later hours of treatment with some anticancer drugs, the cells displayed signs of necrosis with loss of both ECFP/EYFP fluorescence, subsequent to FRET loss. Imaging with large number of compounds revealed that most of the cells shift from apoptotic to necrotic stage, with an average interval of 45 min to 3 h after caspase activation. This clearly reveals that an imaging interval of 30–45 min is sufficient to distinguish the primary necrotic and secondary necrotic cells.

### HTS live cell method for apoptosis and necrosis

**Figure 4:**
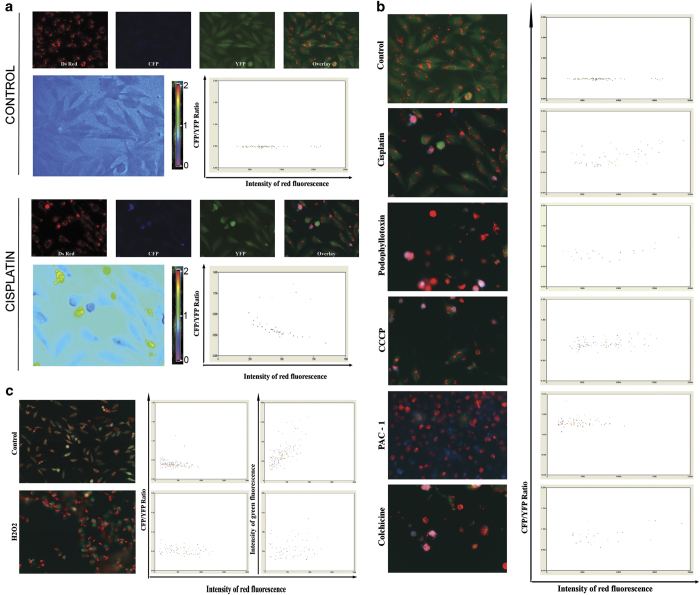
(**a**) U251 DEVD Mito Ds cells were grown on 96-well glass-bottom plates and treated with different drugs. High-throughput imaging for ratio and Mito-DsRed was carried out as described using pathway bioimager. A representative montage image of ECFP–EYFP Ratio and a merged channel of ECFP/EYFP/DsRed and scatter plot generated after proper segmentation and analysis for untreated control and cisplatin (50 *μ*g/ml)-treated samples is shown. (**b**) The merged images and scatter plot from high-throughput imager for cells treated with indicated drugs are shown. (**c**) U251 DEVD Mito Ds cells grown in 96-well glass-bottom plates were treated with necrosis-inducing agent, H_2_O_2_ (2.5 mM). Merged image and scatter plot from high-throughput imager for treated and control cells are shown.

A major application of apoptosis–necrosis detection method is in preclinical cancer drug screening. Even though the above-described approach is sensitive to distinguish necrosis from apoptosis in real-time mode, for compound screening the approach needs to be adapted for high-throughput mode. We have previously described a method using high-throughput imager BD Pathway 435 Bio-imager (BD Biosciences, San Jose, CA, USA) for quantitative caspase activation detection. We have utilized a similar approach with further modification to image the mitochondria and validated for large-scale compound screening. The cells after segmentation were analyzed for ratio change against Mito-DsRed fluorescence. The donor, acceptor, Mito-DsRed and ratio images from untreated control and cisplatin-treated well are shown in Figure 4a along with scatter plot. As seen from the ratio image and scatter plots, the quantitative apoptosis and necrosis is quite evident. Representative merged images and scatter plot of cells treated with podophyllotoxin, CCCP, PAC1, colchicine is shown is Figure 4b. Similarly, the scatter plot and the acceptor intensity of the necrosis induced by H_2_O_2_ is shown in Figure 4c. As seen, there is no donor or acceptor fluorescence in the H_2_O_2_-treated wells; however, such cells can be identified and analyzed on the basis of red mitochondrial fluorescence. Overall, the approach described is highly adaptable for cancer drug screening purpose utilizing HTS imager and can efficiently discriminate between the two forms of cell death.

### Flow cytometry detection and correlation with Annexin-V binding assay

**Figure 5:**
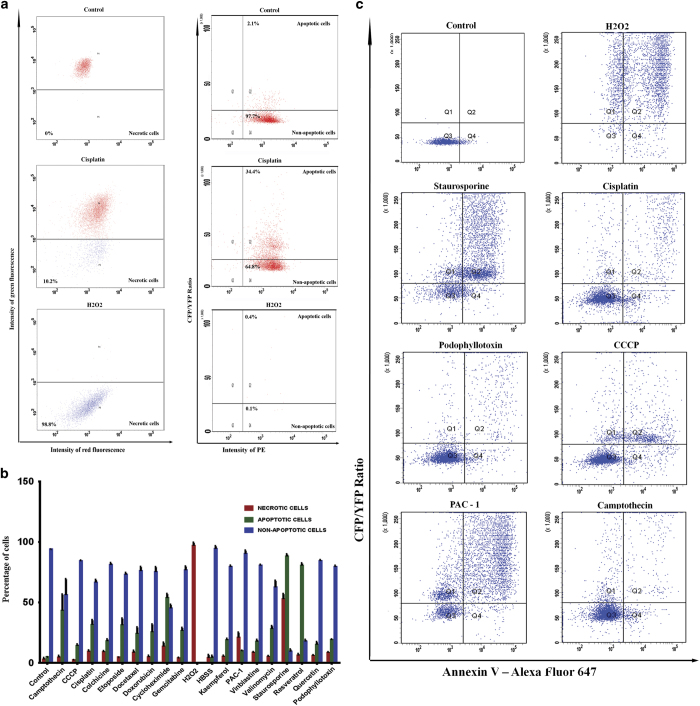
(**a**) U251 DEVD Mito Ds cells were exposed to different drugs for 24 h. The cells were analyzed using flow cytometer for green fluorescence at 488 nm and ratio mode for FRET at 405 nm. The necrotic cells are gated based on Mito-DsRed fluorescence against green channel. The cell with increase in ratio represents cells with caspase activation. (**b**) The percentage of cells with apoptosis and necrosis were scored from the three different flow cytometry data for the indicated drugs. Results represented are mean±S.D. (*n*=3). (**c**) U251 DEVD Mito Ds cells were exposed to different drugs for 24 h. The cells were trypsinized and stained with Alexa Fluor 647 conjugated Annexin-V as described. The cells were analyzed for ratio change and annexin-V staining. The scatter plot of annexin-V *versus* ratio of the cells treated with indicated drugs are shown.

The developed tool is robust and is adaptable for quantitative apoptosis and necrosis detection using flow cytometry. As in microscopy, the cells were excited with the donor ECFP at 405 nm laser line and emission was collected using separate filter sets for ECFP and FRET channel. Similarly, DsRed was excited with green yellow laser line (562 nm) and emission was collected using 590–620 nm filter sets. Further, the cells were also excited with 488 nm to identify the cells with FRET probe to score necrotic activity based on its complete loss. Representative scatter plot of red emission intensity against FRET ratio for untreated and drug-treated cells is shown in Figure 5a. As shown, necrotic cells are identified on the basis of the green fluorescence intensity against red mitochondrial fluorescence. Correlating to the microscopy and HTS imaging results, three different populations are visible representing necrotic, apoptotic and viable fractions. A graph representing the percentage of apoptotic and necrotic cells treated with different drugs is also shown in Figure 5b (corresponding scatter plots are shown in Supplementary Figure 2A and B). Similar results were obtained with Annexin-V staining, which reveals a high correlation of the imaging tool with the currently available cell death assays (Figure 5c).

The results described above confirm the multiplatform compatibility of the assay protocol for distinguishing necrosis and apoptosis in steady-state level and also to conclusively identify the nature of cell death in real-time mode.

## Discussion

The cellular events involved in the programmed demise of a cell and its regulators are well defined. The two forms of apoptosis signaling namely intrinsic and extrinsic pathways ensures peaceful elimination of cells through the concerted activation of cysteine proteases called caspases.^17^ In the intrinsic pathway of apoptosis conformationally activated Bax/Bak induces mitochondrial release of intermembrane space proteins such as cytochrome *c* that immediately initiates the activation of procaspase 9 to caspase 9.^18^ Thus caspase 9 acts as an initiator caspase that subsequently activates downstream caspases such as caspase 7 and 3, and dismantles the cell through cleavage of large number of structural and functional proteins.^19^ In the extrinsic pathway, activation of death receptor culminates in the activation of initiator caspase such as caspase 8 and caspase 10. This subsequently activates downstream caspases, engaging the mitochondria through Bid cleavage followed by its translocation to mitochondria.^20^

The other form of cell death called necrosis is a rapid nonspecific form of cell death, which often kills the cancer cell without involvement of any of the signaling described for apoptosis. It involves the sudden release of toxic proteases and other soluble cellular components into the extracellular environment.^21^ Because of its deleterious consequences to the normal tissues, this form of cell death is considered as undesirable even for cancer chemotherapy. Owing to the importance of both forms of cell death in disease processes and their therapeutic value, diverse methods were described for detection of apoptosis, necrosis and also for simultaneous detection of both. The most popular assay described for discriminating apoptosis from necrosis is Annexin-V PI staining. This method is based on the exposure of phosphatidyl serine (PS) on the surface of the apoptotic cells, which can be detected with the fluorescent conjugated Annexin-V that show high binding affinity to PS. Co-staining of cells with propidium iodide (PI), a cell impermeable nuclear dye, allows identifying cells with loss of membrane permeability, a hallmark of necrosis. Even though it is most popular, this method suffers from several weakness and it is difficult to accurately measure the cells that died of apoptosis and necrosis in end-stage detection because apoptotic cells also lose membrane permeability in the later stages. Similarly, apoptotic cells at later stages lose the ability to bind to Annexin-V because of severe membrane damage. Further, Annexin-V exposure is not exclusive to apoptotic cells as it has been shown to be present in necrotic cells.^22^ Simultaneous analysis of caspase activation and Annexin-V staining brings to light the fact that most necrotic cells showed Annexin-V binding even in the absence of caspase activation. This confirms the inferiority of Annexin-V assay over the current method in discriminating apoptotic from and necrotic cells.

The release of cytosolic enzymes such as lactate dehydrogenase (LDH)^23^ and full-length intact cytokeratin 18 (a high mobility protein), are used extensively to identify necrotic cells.^24^ Even though these approaches are sensitive to detect necrosis, interpretation of results could be ambiguous as post-apoptotic cells also release cytosolic components; if not immediately engulfed by macrophages.

Mitochondrial membrane permeability and caspase activation are the signature events of apoptosis that is absent in necrotic cells. Likewise, apoptotic cells do not exhibit loss of membrane permeability before caspase activation. On the basis of this rationale, we decided to generate a live cell tool to discriminate apoptosis and necrosis. Previous studies from our group have reported the potential of FRET-based approach in real-time detection of caspase activation.^15^ The ECFP-DEVD–EYFP FRET probe is soluble in nature, hence we hypothesized that this probe will be released out from the cell in necrotic cells. Mitochondrial targeted DsRed, which is a relatively photostable and insoluble fluorescent protein,^25,26^ when used in conjunction with the FRET probe, could enhance the predictive power of this imaging tool. Here, we describe the potential application of this tool in discriminating apoptosis and necrosis in real-time mode by microscopy and flow cytometry. A high-throughput image-based approach is also described for easy quantification of apoptosis and necrosis so that large-scale compound screening is also possible in a rapid manner.

The imaging tool, illustrated in this study is easily adaptable to any cell line and is accurate in its discriminating capability. Thus, this assay is the first approach that allows one to discriminate live cells, apoptotic cells and necrotic cells. Existing procedures for differentiating apoptosis and necrosis requires elaborate processing including fixation and use of multiple dyes. In view of this fact, our approach is live cell compatible, needs no further processing and can evaluate drug activity in a dynamic manner. The modified form of annexin staining, called Psiva (polarity sensitive indicator of viability and apoptosis) possess several advantages over conventional Annexin-V staining. However, being a dye-based approach, it is difficult to track apoptotic events for longer time periods. It also suffers from typical drawbacks of annexin staining such as lack of specificity and identification of secondary necrotic cells. Using this assay, we demonstrate that some apoptotic cells transform into necrotic phenotypes, with a delay of 30 min to 3 h. On the basis of this calculation, repeated imaging at an interval of 30 min can differentiate these events at single-cell level even in high-throughput imaging platforms.

A major disadvantage of this assay is the requirement of transfection and stable introduction of the sensors into the specific cells with homogenous expression of both the transgene. In addition, for confirmatory studies to distinguish primary apoptosis-inducing agents from primary necrosis-inducing agent, the assay needs to be performed in a real-time mode. Because of this, it is difficult to adapt this method for normal primary cells and difficult to transfect cells. However, the popular assays such as annexin-V biding or immunodetection of cleaved caspase 3 can be used in any cell of choice. Similarly, studies requiring better temporal resolution of entry into apoptosis and its progression at single-cell level in real-time mode might require more complex imaging conditions with constant fast recording possibilities.

However, to some extent, use of better methods of stable transgene introduction into primary cells and difficult to transfect cells such as lentiviral or retroviral gene delivery can address few of the above-described drawbacks. Similarly, once the stable cells are developed, they form renewable cell resources for pathway-defining or drug-screening applications. Even though the results from only one cell is described here, currently, we are generating stable cells expressing these probes in selected NCI cancer cell line panels to be used as next-generation preliminary cancer drug-screening platform in high-throughput mode.

**Figure 6:**
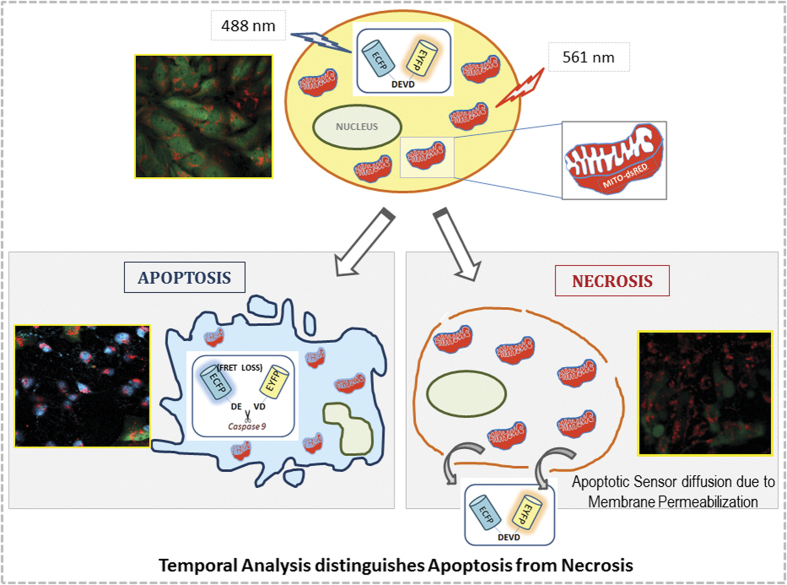
The schema summarizes the potentiality of the tool to discriminate the two forms of cell death: apoptosis and necrosis in a spatio-temporal manner. The apoptotic cells appear blue due to FRET loss, at the same time retaining the insoluble Mito-DsRed probe; whereas, in necrotic cells, the FRET probe is lost due to membrane permeabilization and are characterized by the presence of insoluble Mito-DsRed probe alone. The healthy cells retain both the intact FRET probe and the Mito-DsRed probe

In summary, the method described here (Figure 6) will form a new robust, easy-to-perform approach to confirm the nature of cell death; with potential application in cancer drug screening and understanding the mechanistic insight of drug action. Being a real-time compatible and confirmatory approach, it could have a significant role in understanding the regulators of apoptosis and necrosis with high sensitivity at single-cell level.

## Materials and Methods

### Cells, cell lines and maintenance

Human neuroblastoma cell U251 cell line was procured from NCI and used for the experiments within the first five passages after revival. The cells were maintained in DMEM medium (Invitrogen, Carlsbad, CA, USA) supplemented with 10% FBS (Sigma-Aldrich Inc, St. Louis, MO, USA).

### Chemicals and reagents

The drugs used for the study are listed in the Supplementary Table 1 with their effective concentration and known mechanism of action. The chemical compounds were obtained from Santa Cruz Biotechnology, Santa Cruz, CA, USA, Sigma-Aldrich Inc, St. Louis, MO, USA or Calbiochem, San Diego, CA, USA.

### Expression vectors and transfections

The following expression vectors were used for generating stable cell lines. The expression vector pcDNA3 ECFP-DEVD-EYFP (caspase sensor FRET probe) was a gift from Professor Jeremy M Tavere^16^ and Professor Gavin Welsh (University of Bristol, UK). Expression vector for Mito-DsRed (#6975-1) was purchased from Clontech Laboratories Inc, Mountain View, CA, USA. Initially, the cells were transfected with the pcDNA3 ECFP-DEVD–EYFP expression vector using lipofectamine LTX (Invitrogen, #15338-100) as per the manufacturer’s protocol. Stably expressing clones were generated by selecting the cells in 800 *μ*g/ml of G418 (Invitrogen) containing medium for 30–40 days. Single-cell clones were expanded and used for further experiments. The stable U251 Caspase sensor cell was further transfected with Mito-DsRed. The cells expressing both FRET probe and Mito-DsRed were sorted on the basis of EYFP fluorescence and DsRed fluorescence using FACSAria III. Multiple clones were expanded and the clones that stably maintained homogeneous level of both the probes were used for all the subsequent experiments.

### Ratio imaging by microscopy and high-throughput imaging

For ratio imaging, the cells were grown in chambered cover glass (Lab-TekTM, Nunc, NY, USA) at the desired density and were treated with the drugs. For microscopic ECFP/EFYP FRET ratio imaging, the cells were excited with excitation filter of 438±24 nm and dual emission was collected with 483±32 nm and 542±27 nm using the dichroic 458LP. DsRed was viewed through a filter combination of Ex: 545±30, Em: 620±60 and dichroic 570LP. The excitation and emission filter wheels were independently controlled through IPLab software interlinked through CARV confocal unit. The images were acquired using the camera CoolSnapHQ2 (Photometrics, Surrey, British Columbia, Canada) equipped in Ti microscope (Nikon, Japan).

For imaging cells in 96-well plates, the cells were seeded in 96-well glass-bottom plates (Greiner Bio-One GmbH, KremsmÃ¼nster, Austria) at the desired density. After 24 h, the medium was removed and replenished with 5% FBS-supplemented DMEM containing the drugs at various concentrations. The plates were imaged under Pathway Bio-imager 435 (BD Biosciences, San Jose, CA, USA) as described earlier.^15^ The images of cells in each well were acquired in the respective channels using a dry ×20 objective with NA 0.7. The images were captured as 2×2 montages integrated as a single image for each well. After acquisition, the cells were segmented on the basis of mitochondrial fluorescence channel, and the ratio and intensity of DsRed were used for generating scatter plots for all the wells.

### Confocal imaging of FRET and Mito-DsRed

The stably expressing cells were grown in chambered cover glass or 96-well glass-bottom plates at 40–60% confluency. The cells were treated with different drugs. All confocal imaging was carried out using Nikon A1R confocal microscope. The FRET probe was excited with 456 nm laser line from multiline Argon source. The dual emission 465–500 nm for ECFP and 525–555 for EYFP was collected simultaneously with two independent PMTs and analyzed in ratio mode. The PMT voltage was adjusted to maintain an average ratio of 0.5 in untreated control. Once standardized with control, the same image parameters were maintained for all the treated samples. Mito-DsRed was excited with 562 nm laser line and emission at 570–620 was collected using separate PMT. All confocal images were analyzed using NIS Element in ratio mode simultaneous with DsRed fluorescence for determining apoptosis and necrosis. For real-time imaging purpose, the cells were continuously maintained on stage supported with a well plate incubator from Okolab (Pozzuoli NA, Italy) for maintaining 37 °C and 5% CO_2_. For few time lapse imaging, the multipoints were memorized using the NIS element software and the same XYZ plane was repeatedly imaged at different time points. The plates were regularly maintained in conventional CO_2_ incubator.

### Flow cytometer determination of apoptosis and necrosis using ratio mode flow cytometry

Flow cytometry analysis was done using FACSAria III equipped with 405nm, 488nm and 561 nm, 633 nm laser line. The single-cell suspension of cells were filtered through 30 *μ*m cell strainer and immediately analyzed. The filter combination used for FRET analysis include two filters configured in the 405 laser line 450/50 and 48/25. For detecting DsRed, a filter sets of 582/15 was configured in the yellow green laser line, 561nm path. The dual emission at 465–500 nm for ECFP and 525–555 for EYFP was collected in 405 nm laser path and analyzed in ratio mode with a multiplication factor of 250 simultaneous with collection of red fluorescence for DsRed. The FRET probe was also excited with 488 nm laser for detecting the signal at 530/30 so that all the cells expressing the FRET probe can be identified primarily to distinguish the negative necrotic cells.

### Comparison of caspase assay against Annexin-V binding using flow cytometer

The cells were exposed to different drugs and after indicated treatment periods, the cells were trypsinized and stained with Alexa fluor 647 conjugated with Annexin-V (Molecular probes, USA) in binding buffer for 20 min as per the standard protocol. The cells were strained using 30 *μ*m cell strainer and immediately analyzed using flow cytometer for caspase activation in ratio mode as described above. The emission of Alexa fluor 647 was collected at 660/20 channel exciting with 633 nm laser. For discriminating apoptosis from necrosis fluorescence emission at 530/30 was also collected by exciting with 488 nm laser line. Necrosis is scored if the EYFP fluorescence drops below the threshold value set based on control fluorescence value.

## Notes

Supplementary Figure S1

Supplementary Figure S2A

Supplementary Figure S2B

Supplementary Video 1A

Supplementary Video 1B

Supplementary Video 2

Supplementary Video 3A

Supplementary Video 3B

Supplementary Video 4A

Supplementary Video 4B

Supplementary Video 5A

Supplementary Video 5B

## References

[bib1] Hetts SW. To die or not to die: an overview of apoptosis and its role in disease. JAMA 1998; 279: 300–307. 9450715 10.1001/jama.279.4.300

[bib2] Thompson CB. Apoptosis in the pathogenesis and treatment of disease. Science 1995; 267: 1456. 7878464 10.1126/science.7878464

[bib3] Kitada S, Andersen J, Akar S, Zapata JM, Takayama S, Krajewski S et al. Expression of apoptosis-regulating proteins in chronic lymphocytic leukemia: correlations with *in vitro* and *in vivo* chemoresponses. Blood 1998; 91: 3379–3389. 9558396

[bib4] Johnstone RW, Ruefli AA, Lowe SW. Apoptosis: a link between cancer genetics and chemotherapy. Cell 2002; 108: 153–164. 11832206 10.1016/s0092-8674(02)00625-6

[bib5] Devarajan E, Sahin AA, Chen JS, Krishnamurthy RR, Aggarwal N, Brun AM et al. Down-regulation of caspase 3 in breast cancer: a possible mechanism for chemoresistance. Oncogene 2002; 21: 8843–8851. 12483536 10.1038/sj.onc.1206044

[bib6] Brouckaert G, Kalai M, Krysko DV, Saelens X, Vercammen D, Ndlovu MN et al. Phagocytosis of necrotic cells by macrophages is phosphatidylserine dependent and does not induce inflammatory cytokine production. Mol Biol Cell 2004; 15: 1089–1100. 14668480 10.1091/mbc.E03-09-0668 PMC363082

[bib7] McCarthy NJ, Evan GI. 15 Methods for detecting and quantifying apoptosis. Curr Topics Dev Biol 1997; 36: 259–278. 10.1016/s0070-2153(08)60507-4 9342533

[bib8] Vermes I, Haanen C, Steffens-Nakken H, Reutellingsperger C. A novel assay for apoptosis flow cytometric detection of phosphatidylserine expression on early apoptotic cells using fluorescein labelled annexin V. J Immunol Methods 1995; 184: 39–51. 7622868 10.1016/0022-1759(95)00072-i

[bib9] Sawai H, Domae N. Discrimination between primary necrosis and apoptosis by necrostatin-1 in Annexin V-positive/propidium iodide-negative cells. Biochem Biophys Res Commun 2011; 411: 569–573. 21763280 10.1016/j.bbrc.2011.06.186

[bib10] Hirsch T, Marchetti P, Susin SA, Dallaporta B, Zamzami N, Marzo I et al. The apoptosis-necrosis paradox. Apoptogenic proteases activated after mitochondrial permeability transition determine the mode of cell death. Oncogene 1997; 15: 1573–1581. 9380409 10.1038/sj.onc.1201324

[bib11] Lemasters JJ. V Necrapoptosis and the mitochondrial permeability transition: shared pathways to necrosis and apoptosis. Am J Physiol Gastrointest Liver Physiol 1999; 276: G1–G6. 10.1152/ajpgi.1999.276.1.G1 9886971

[bib12] Henry-Mowatt J, Dive C, Martinou JC, James D. Role of mitochondrial membrane permeabilization in apoptosis and cancer. Oncogene 2004; 23: 2850–2860. 15077148 10.1038/sj.onc.1207534

[bib13] Anghileri LJ, Robert J. Effects of tumor necrosis factor on tumor cell plasma membrane permeability. Tumori 1987; 73: 269–271. 3603723 10.1177/030089168707300310

[bib14] Ewunkem AJ, Parson CD, Muganda PM, Newman RH. A low-cost method for tracking the induction of apoptosis using FRET-based activity sensors in suspension cells. Apoptosis Methods Toxicol 2016, pp 93–108.

[bib15] Joseph J, Seervi M, Sobhan PK, Retnabai ST. High throughput ratio imaging to profile caspase activity: potential application in multiparameter high content apoptosis analysis and drug screening. PLoS One 2011; 6: e20114. 21637712 10.1371/journal.pone.0020114 PMC3103529

[bib16] Tyas L, Brophy VA, Pope A, Rivett AJ, Tavare JM. Rapid caspase-3 activation during apoptosis revealed using fluorescence-resonance energy transfer. EMBO Rep 2000; 1: 266–270. 11256610 10.1093/embo-reports/kvd050 PMC1083724

[bib17] Kiraz Y, Adan A, Kartal Yandim M, Baran Y. Major apoptotic mechanisms and genes involved in apoptosis. Tumour Biol 2016; 37: 8471–8486. 27059734 10.1007/s13277-016-5035-9

[bib18] Martinou JC, Youle RJ. Mitochondria in apoptosis: Bcl-2 family members and mitochondrial dynamics. Dev Cell 2011; 21: 92–101. 21763611 10.1016/j.devcel.2011.06.017 PMC3156409

[bib19] Shalini S, Dorstyn L, Dawar S, Kumar S. Old, new and emerging functions of caspases. Cell Death Differ 2015; 22: 526–539. 25526085 10.1038/cdd.2014.216 PMC4356345

[bib20] Fulda S. Caspase-8 in cancer biology and therapy. Cancer Lett 2009; 281: 128–133. 19111387 10.1016/j.canlet.2008.11.023

[bib21] Krysko DV, Vanden Berghe T, D'Herde K, Vandenabeele P. Apoptosis and necrosis: detection, discrimination and phagocytosis. Methods 2008; 44: 205–221. 18314051 10.1016/j.ymeth.2007.12.001

[bib22] Atale N, Gupta S, Yadav UC, Rani V. Cell-death assessment by fluorescent and nonfluorescent cytosolic and nuclear staining techniques. J Microsc 2014; 255: 7–19. 24831993 10.1111/jmi.12133

[bib23] Chan FK, Moriwaki K, De Rosa MJ. Detection of necrosis by release of lactate dehydrogenase activity. Methods Mol Biol 2013; 979: 65–70. 23397389 10.1007/978-1-62703-290-2_7 PMC3763497

[bib24] Linder S, Havelka AM, Ueno T, Shoshan MC. Determining tumor apoptosis and necrosis in patient serum using cytokeratin 18 as a biomarker. Cancer Lett 2004; 214: 1–9. 15331168 10.1016/j.canlet.2004.06.032

[bib25] Verkhusha VV. An enhanced mutant of red fluorescent protein DsRed for double labeling and developmental timer of neural fiber bundle formation. J Biol Chem 2001; 276: 29621–29624. 11408473 10.1074/jbc.C100200200

[bib26] Wiehler J, von Hummel J, Steipe B. Mutants of Discosoma red fluorescent protein with a GFP-like chromophore. FEBS Lett 2001; 487: 384–389. 11163363 10.1016/s0014-5793(00)02365-6

