# Supplementary material for: A quantitative real-time approach for discriminating apoptosis and necrosis
Source: Cell Death Discov. 2017 Jan 23;3:16101–. doi: 10.1038/cddiscovery.2016.101 (PMC5253725; doi:10.1038/cddiscovery.2016.101)
Supplement: Supplementary Table S1 [file cddiscovery2016101-s1.doc]

# SUPPLEMENTARY TABLE 1

**Details of drugs used for screening in the study**

| **Sl.**  **no.** | **Compound** | **Possible Mechanism of action** | **Working concentration** |
| --- | --- | --- | --- |
| **1** | Camptothecin | Irreversibly binds to the topoisomerase I, inhibiting DNA reassociation | 1 µM |
| **2** | Carbonyl cyanide m-chlorophenyl hydrazone (CCCP) | A protonophore, inhibits oxidative phosphorylation | 5 µM |
| **3** | Cisplatin | Cross-links DNA | 50 µg/ml |
| **4** | Colchicine | Microtubule inhibitor | 50 µg/ml |
| **5** | Cycloheximide | Translation inhibitor | 100 µg/ml |
| **6** | Docetaxel | Binds to microtubules | 2 µM |
| **7** | Doxorubicin | Reverse transcriptase and RNA Pol inhibitor, DNA intercalator | 200 ng/ml |
| **8** | Etoposide | Topoisomerase inhibitor | 25 µM |
| **9** | Kaempferol | Nuclear DNA degradation, lipid peroxidation, Inhibits Topi –I, catalyzed DNA relegation | 100 µM |
| **10** | Gemcitabine | Nucleoside analog | 50 µM |
| **11** | H_2_O_2_ | Generates ROS | 2.5mM |
| **12** | PAC-1 | Procaspase Activating Compound | 50 µM |
| **13** | Podophyllotoxin | Inhibits microtubule assembly | 10 µM |
| **14** | Quercetin | Activates apoptotic cascade | 50 µM |
| **15** | Resveratrol | Phenolic antioxidant, COX-1 inhibitor | 50 µM |
| **16** | Staurosporine | Protein kinase inhibitor | 500 nM |
| **17** | Taxol | Inhibits microtubule disassembly, blocks Bcl-2 | 2 µM |
| **18** | Valinomycin | HDAC inhibitor /Ionophore | 5 µM |
| **19** | Vinblastine | Microtubule inhibitor | 100nM |
