# Supplementary figures and images for: A quantitative real-time approach for discriminating apoptosis and necrosis
Source: Cell Death Discov. 2017 Jan 23;3:16101–. doi: 10.1038/cddiscovery.2016.101 (PMC5253725; doi:10.1038/cddiscovery.2016.101)

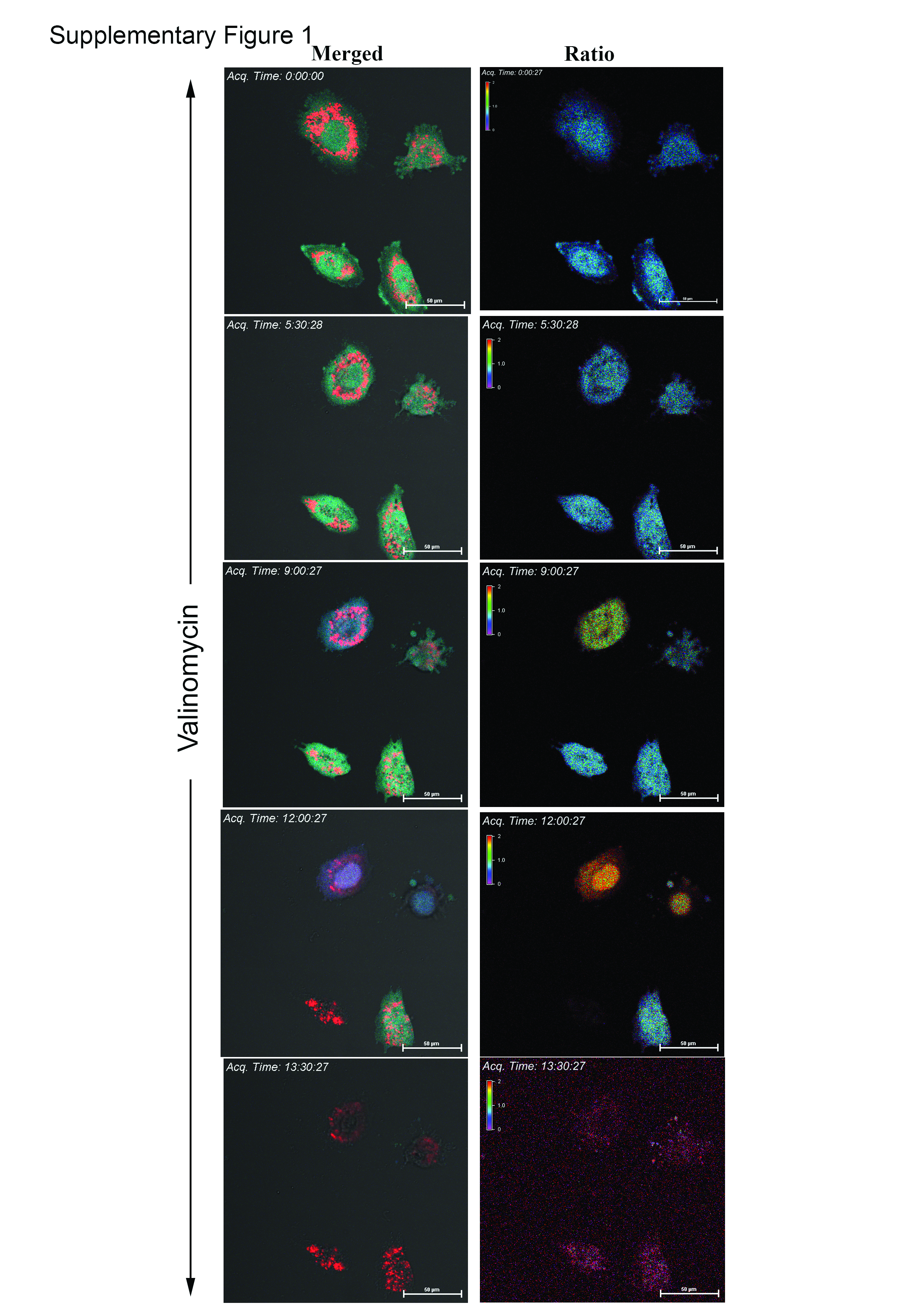

Supplement: Supplementary Figure S1 [file cddiscovery2016101-s2.tiff]

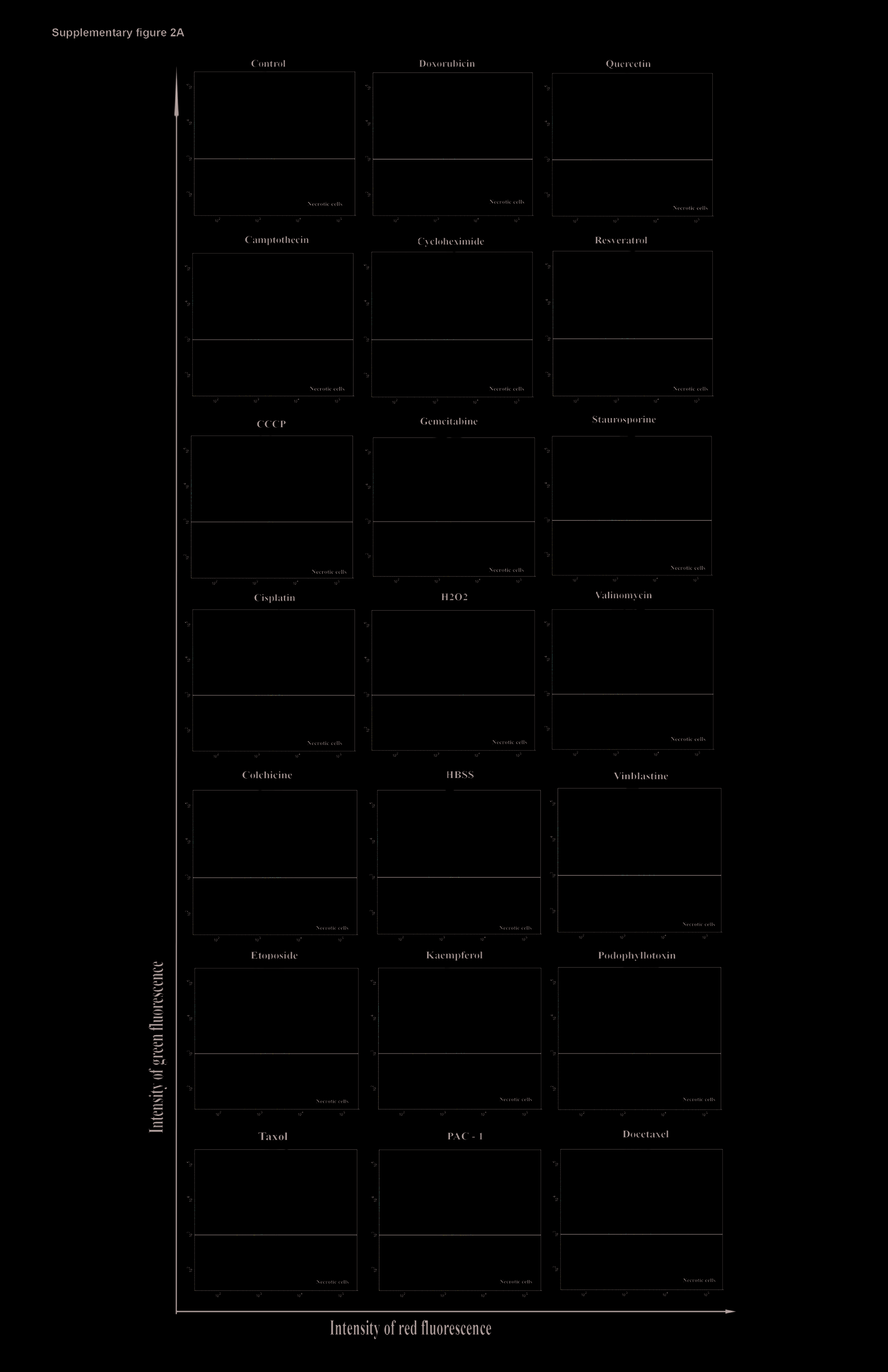

Supplement: Supplementary Figure S2A [file cddiscovery2016101-s3.tiff]

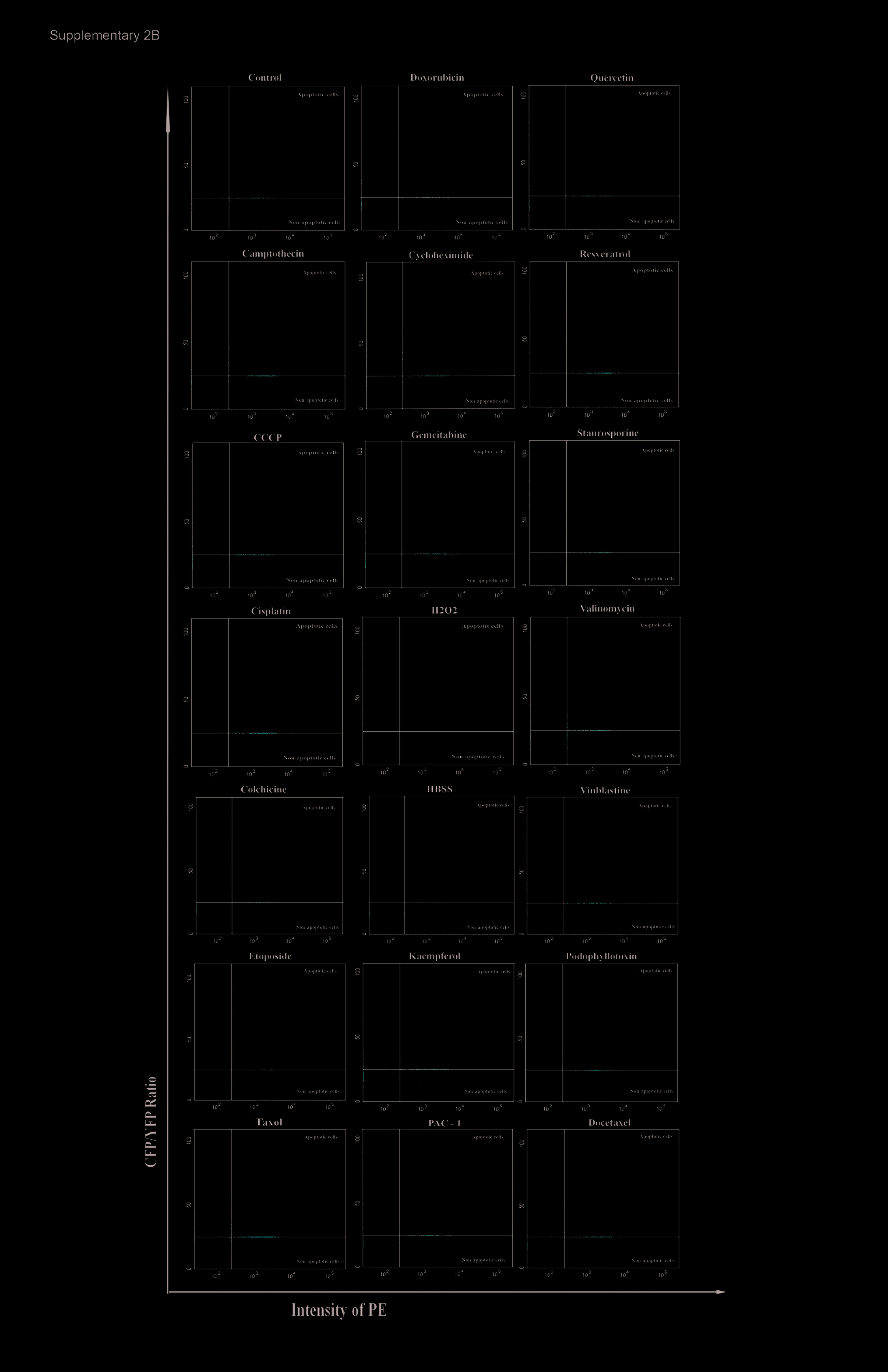

Supplement: Supplementary Figure S2B [file cddiscovery2016101-s4.tiff]
